# Uptake, retention, and outcomes in a demonstration project of pre-exposure prophylaxis among female sex workers in public health centers in Senegal

**DOI:** 10.1177/0956462420943704

**Published:** 2020-08-20

**Authors:** Moussa Sarr, Daouda Gueye, Aminata Mboup, Ousmane Diouf, Mame D Bousso Bao, Anna Julienne Ndiaye, Birahim P Ndiaye, Stephen E Hawes, Eric Tousset, Abdoulaye Diallo, Fatima Jones, Coumba T Kane, Safiatou Thiam, Cheikh T Ndour, Geoffrey S Gottlieb, Souleymane Mboup

**Affiliations:** 1 Westat, Inc., Rockville, MD, USA; 2 IRESSEF: Institut de Recherche en Santé de Surveillance Epidémiologique et de Formations, Dakar, Sénégal; 3 Departments of Epidemiology and Global Health, School of Public Health, University of Washington, Seattle, WA, USA; 4 Aardex Group, Vise, Belgium; 5 Faculte de Médicine et de Pharmacie, Université Cheikh Anta Diop, Dakar, Sénégal; 6 Conseil national de lutte contre le sida (CNLS), Dakar, Sénégal; 7 Division de Lutte contre le Sida et les IST, Ministère de la Santé et de l’Action Sociale, Dakar, Sénégal; 8 Department of Medicine, Allergy and Infectious Diseases, Center for Emerging and Re-Emerging Infectious Diseases and Department of Global Health, University of Washington, Seattle, WA, USA

**Keywords:** Africa, sex workers, HIV, prevention, women

## Abstract

The Senegal pre-exposure prophylaxis (PrEP) Demonstration Project was an open-label cohort study assessing the delivery of daily oral PrEP to HIV-negative female sex workers (FSWs) in four Ministry of Health (MoH)-run clinics in Dakar, Senegal. We assessed uptake, retention in care, and adherence over up to 12 months of follow-up as well as HIV infection rates. Between July and November 2015, 350 individuals were approached and 324 (92.6%) were preliminarily eligible. Uptake was high, with 82.4% of eligible participants choosing to enroll and take PrEP. The mean age of those enrolled was 37.7 years (SD = 8.7), and approximately half had not attended school (41.2%). Among the 267 participants who were prescribed PrEP, 79.9 and 73.4% were retained in PrEP care at 6 and 12 months, respectively. Older age among FSWs was found to be the only significant predictor of lower discontinuation. We did not find significant differences in retention by site, education, condom use, or HIV risk perception. There were no new HIV infections at follow-up. Our results showed evidence of high interest in PrEP and very good PrEP retention rates among FSWs at 12-month follow-up when offered in MoH-run clinics, with older age as the only significant predictor of higher PrEP retention. This highlights the role that these clinics can play in expanding PrEP access nationwide.

## Introduction

Antiretroviral (ARV)-based prevention of HIV transmission has the potential to have a profound population-level impact on the course of the HIV/AIDS pandemic. Several completed randomized controlled trials of HIV pre-exposure prophylaxis (PrEP) have shown efficacy at reducing HIV acquisition in high-risk populations of men who have sex with men (MSM), transgender women,^1^ HIV sero-discordant partners,^2^ high-risk heterosexual adults,^3^ and injection drug users^4^; however, not all studies have shown efficacy in high-risk populations (e.g. the VOICE and FEM-PrEP trials).^5–7^ What has become clear from these studies is that adherence to daily PrEP is key to providing benefit and reducing incident HIV infections.^8,9^ Although all the aforementioned clinical trials provide strong evidence that PrEP can work in preventing HIV acquisition in a controlled clinical trial setting, there is less evidence that HIV PrEP can be implemented as part of a public health approach through country-level ministries of health (MoH) as part of a combination package of HIV prevention services to high-risk populations in resource-limited settings. HIV PrEP demonstrations projects may provide data on the feasibility of implementation and potential for sustainability in routine programmatic settings. Recently, results from PrEP demonstration projects mostly conducted in East and Southern Africa have been reported with varying results.^7,10^

Senegal in West Africa has maintained a low prevalence of less than 1% HIV in the general population and has run an effective HIV prevention program.^11^ Sex work is legal and regulated in Senegal following a 1969 law.^12^ According to the law, all self-identified female sex workers (FSWs), at least 21 years old, must be registered in health clinics and come to the government-run health clinics to receive monthly checkups, free condoms, education on sexually transmitted infection (STI) and contraceptives, and prescriptions for medication as needed. Sex workers are also screened every two months for gonorrhea, chlamydia, trichomoniasis, and other vaginal bacterial infections. Serological screening is conducted twice a year to test for syphilis and once a year to test for HIV status. The ‘unregistered’ sex workers also have access to prevention and care services when they come to the clinics but these services are not delivered due to lack of registration. The ‘unregistered’ FSWs who do not want to have a ‘registration card’ can be subject to the consequences of ‘breaking the law’ by the police when practicing sex work. However, despite decades of regulation of sex work and related HIV prevention programs, the HIV prevalence in FSWs in Senegal remains unacceptably high, with prevalence rates ≥7%.^13–16^ Thus, novel prevention strategies in FSWs, such as PrEP, are urgently needed. This PrEP Demonstration Project in Senegal was designed to support the integration of oral PrEP (emtricitabine/tenofovir disoproxil fumarate [FTC/TDF], Truvada, Gilead Sciences, Foster City, CA, USA),^17^ as part of the existing HIV prevention package received by FSWs in dedicated MoH-run FSW clinics in Rufisque, Diamniadio, Pikine, and Mbao Health Centers.

The objective of the study was to demonstrate the feasibility of providing daily oral PrEP with FTC/TDF for 12 months to FSWs at MoH-run clinics in Senegal.

## Methods

### Study design and participants

The Senegal Demonstration Project (clinicaltrials.gov # NCT02474303) was a prospective, open-label cohort study assessing the delivery of daily oral FTC/TDF PrEP integrated into the HIV prevention package of FSWs in four government-run clinics in Dakar. This is within a context of regulated sex work, where women ≥21 years of age can officially register as FSWs in a National Public Health registry and be issued a health card. However, for this study, efforts were made to enroll both registered and unregistered sex workers into PrEP.

PrEP was implemented in real-world clinical settings, in four MoH-run clinics in the suburbs of Dakar, Senegal: in the Pikine, Mbao, Rufisque, and Diamniadio Health Centers.

Eligible women were ≥18 years of age, active registered and unregistered sex workers (reported paid sex within the past six months), HIV-negative, and residents of Dakar, Senegal.

At the time of enrollment, participants had to be HIV-1 and HIV-2 seronegative with no signs or symptoms of acute HIV infection, have normal renal function (defined as an estimated creatinine clearance ≥60 ml/min using the Cockcroft–Gault equation with ideal body weight), and not be pregnant or breastfeeding. Finally, we closely followed those with a positive hepatitis B surface antigen (HBsAg) for repeated hepatitis B (HBV) viral load (VL) testing. Those with an HBV VL of greater than 2000 IU/ml, indicating active infection, were excluded from the study and referred to the National Hepatitis B Program for care and treatment.

### Procedures

At the screening visit, after written informed consent was obtained, participants completed evaluations including review of signs and symptoms of potential acute HIV infection, urine β-HCG for pregnancy, HIV-1/HIV-2 Ab/Ag 4th generation testing, HBsAg testing, and serum chemistry including creatinine testing.

The enrollment visit was scheduled to occur within two weeks after the screening visit. Results of the screening lab tests were reviewed to confirm that subjects met all the eligibility criteria. Follow-up visits were scheduled at seven days, 1, 3, 6, 9, and 12 months after enrollment. If a clinical follow-up visit could not be conducted as scheduled, the preferred timeframe for completion of that visit was within seven calendar days prior to or after the target visit date. A one-month supply (30 pills) of the study drug labeled as FTC 200 mg/TDF 300 mg (Truvada) was provided to participants at the enrollment visit. A two-month supply (60 pills) was provided at month 1 and then a three-month supply (90 pills) at quarterly study visits through the month 12 visit. Adherence counseling was routinely provided to all study participants at month 1 at each visit thereafter. Study participants also received free condoms, STI testing and treatment, and appropriate counseling at the enrollment visit and at each study visit thereafter.

### Follow-up lab testing

Laboratory procedures at follow-up visits included urine β-HCG at all visits, urine dipstick for proteinuria and glucosuria at 3, 6, and 12 months and HIV-1/HIV-2 Ab/Ag 4th generation testing at all visits.

Safety lab testing performed at all follow-up visits included serum chemistry (creatinine), liver function tests (aspartate aminotransferase, alanine aminotransferase), and complete blood count with differentials.

In addition, STI screening (Abbott RealTime *Neisseria gonorrhoeae*/*Chlamydia trachomatis* assays, and rapid plasma reagin with *Treponema pallidum* hemagglutination assay confirmation for syphilis) and HBsAg tests were also performed.

### Measures

PrEP uptake, retention in care, and HIV incidence at 12 months are the primary outcomes of this article. Uptake was defined as the proportion of potentially eligible participants who elected to enroll in the study (PrEP uptake). Retention was defined as the number and percentage of patients who stayed in care at 1, 3, 6, 9, and 12 months of follow-up. Participants were considered lost to follow-up when they missed over two consecutive quarterly clinic visits. Participants were considered retained in the program if they had not withdrawn or been lost to follow-up during the 12-month period.

 We assessed PrEP adherence continuously using electronic monitoring (Medical Events Monitoring System 6 [MEMS®], AARDEX Group, Vise, Belgium) throughout the study. The MEMS® cap contains a micro-electronic circuit that records each opening of the pill container. Adherence to PrEP medication was also assessed through blood drug levels (tenofovir [TFV] and FTC) (at 3, 6, 9, and 12 months).^18^

### Statistical analyses

To describe the study population, we generated means (standard deviations) or medians (interquartile range) as needed based on the distribution of continuous variables, and we computed frequencies and simple proportions for categorical variables. Between-group comparisons at baseline were done using Chi square, Fisher’s exact, or *t-*tests as appropriate. PrEP uptake was calculated as the number of participants enrolled divided by the number of potentially eligible clients assessed. Survival analysis (Kaplan–Meier) was used to estimate the time to discontinuation of PrEP. Cox proportional hazard analysis was used to assess demographic characteristics associated with study discontinuation.

Analyses were performed using SAS/STAT® software, Version 9.4 of the SAS System for Windows (SAS Institute Inc, Cary, NC, USA).

### Ethical considerations

Approval to conduct the study was received from the Senegal National Ethics Committee (CNERS), Westat Institutional Review Board (IRB), and from the University of Washington, Seattle IRB. All participants provided written informed consent prior to study participation.

## Results

**Figure 1.**
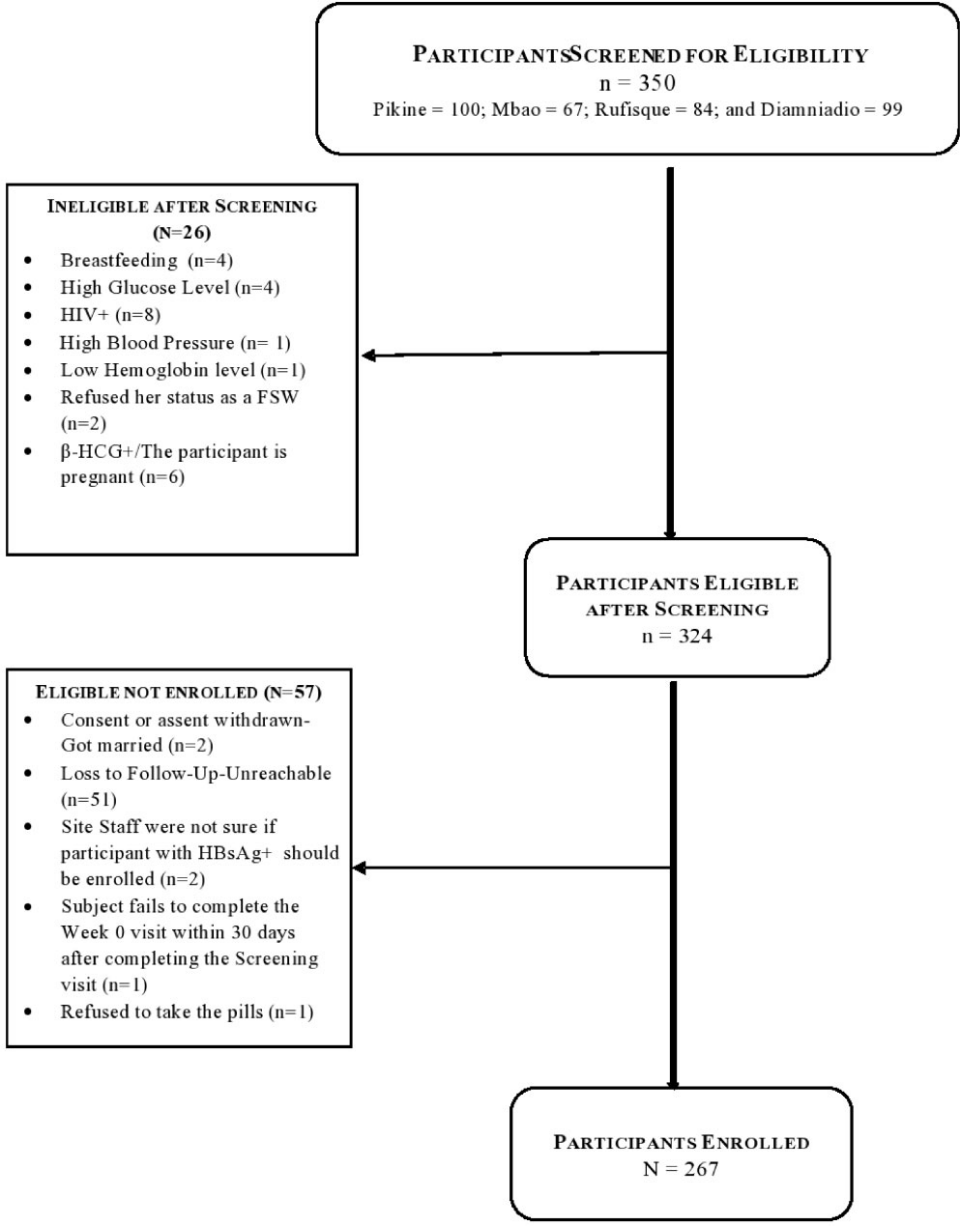
Recruitment process of the PrEP demonstration study. FSW: female sex worker; HBsAg+: positive hepatitis B surface antigen; β-HCG: Beta-Human Chorionic Gonadotropin.

Between July and November 2015, 350 individuals were screened for enrollment, and 324 (92.6%) were preliminarily eligible. At screening eight FSWs had undiagnosed HIV infection and were among those excluded (Figure 1). The HIV prevalence at screening was 2.3% (95% CI: 0.8–4.6), with also a prevalence of positive HBsAg at 8.9% (95% CI: 5.6–12.1). For STIs, the prevalence estimates at screening were syphilis 1.5% (95% CI: 0.6–3.8), chlamydia 6.1% (95% CI: 3.1–12.0), and gonorrhea 4.6% (95% CI: 2.1–9.7).

Among those eligible, 267 participants chose to enroll, leading to a PrEP uptake of 82.4% (Table 1). Unregistered FSWs were significantly more likely to enroll than registered FSWs (RR = 1.16; 95% CI: 1.06–1.26; p = 0.004). Overall, 91.0% (101 out of 111) of eligible unregistered FSWs chose to enroll in PrEP versus 79.4% (170 out of 214) of eligible registered FSWs. We did not find significant differences of uptake by site, age, education, or marital status (data not shown).

**Table 1.** Baseline characteristics of FSWs enrolled into the Senegal PrEP Demonstration Project.

			Health centers/Sites				
Parameter		Overall (n = 267)	Pikine (n = 73)	Mbao (n = 52)	Rufisque (n = 66)	Diamniadio (n = 76)	p-value^µ,^*
Age (years), mean (SD)	Mean (SD)	37.7 (8.7)	36.1 (9.0)	37.7 (7.9)	40.0 (9.0)	37.2 (8.6)	0.19
	Min	18	20	22	18	18	
	Max	57	53	54	57	57	
Registration status	Registered	170 (63.9%)	63 (86.3%)	26 (50%)	47 (71.2%)	34 (45.3%)	<0.01*
	Non-registered	96 (36.1%)	10 (13.7%)	26 (50%)	19 (28.8%)	41 (54.7%)	
Nationality, n (%)	Senegalese	263 (98.5%)	72 (98.6%)	50 (96.2%)	65 (98.5%)	76 (100%)	0.34
	Non-Senegalese	4 (1.5%)	1 (1.4%)	2 (3.8%)	1 (1.5%)	0 (0%)	
Ethnicity, n (%)	Wolof	110 (41.2%)	35 (47.9%)	9 (17.3%)	28 (42.4%)	38 (50.0%)	<0.01*
	Pulaar	68 (25.5%)	20 (27.4%)	16 (30.8%)	12 (18.2%)	20 (26.3%)	
	Sereer	42 (15.7%)	13 (17.8%)	11 (21.2%)	13 (19.7%)	5 (6.6%)	
	Other	47 (17.6%)	5 (6.8%)	16 (30.0%)	13 (19.7%)	13 (17.1%)	
Education	None	110 (41.3%)	35 (48.6%)	25 (48.1%)	18 (27.3%)	32 (42.1%)	
	Primary	133 (50.0%)	25 (34.7%)	25 (48.1%)	43 (65.2%)	40 (52.6%)	<0.01*
	Secondary	23 (8.7%)	12 (16.7%)	2 (3.8%)	5 (7.6%)	4 (5.3%)	
Marital status, n (%)	Single	54 (20.2%)	17 (23.3%)	9 (17.3%)	14 (21.2%)	14 (18.4%)	0.95
	Married	3 (1.1%)	2 (2.7%)	0 (0%)	0 (0%)	1 (1.3%)	
	Separated/Divorced/Widow	210 (78.7%)	54 (74.0%)	43 (82.7%)	52 (78.8%)	61 (80.3%)	
Condom use (during last sex with a client), n (%)	Yes	241 (97.6%)	68 (100%)	46 (97.9%)	57 (98.3%)	70 (94.6%)	0.45
	No	6 (2.4%)	0 (0%)	1 (2.1%)	1 (1.7%)	4 (5.4%)	
	Don’t know/missing	20	5	5	9	2	
Number of clients/last week	Median (IQR)	1 (8.0)	2 (4)	1 (3)	1 (4)	1 (4)	0.49
	(minimum − maximum)	(0–10)	(0–9)	(0–7)	(0–8)	(1–10)	

IQR: interquartile range; PrEP: pre-exposure prophylaxis.

^µ^P-value is from ANOVA or Kruskal–Wallis H test for continuous variables and from Fisher’s exact test or Chi square statistics as appropriate for categorical variables.

*P-value significant at < 0.05.

The average age of those enrolled was 37.7 years (SD = 8.7). Most FSWs were Senegalese (98.5%) and registered sex workers (63.9%). For education, approximately half (41.3%) of the participants never went to school, 50% had attended primary school, and only 8.7% went to high school. Overall, 78.7% of participants described themselves as separated, divorced, or widowed; 20.2% as single; and only 1.1% as married (Table 1).

The median number of clients seen during the previous week and reported by the participants at baseline was 1.0 (interquartile range = 8; minimum = 0 and maximum = 10).

**Figure 2.**
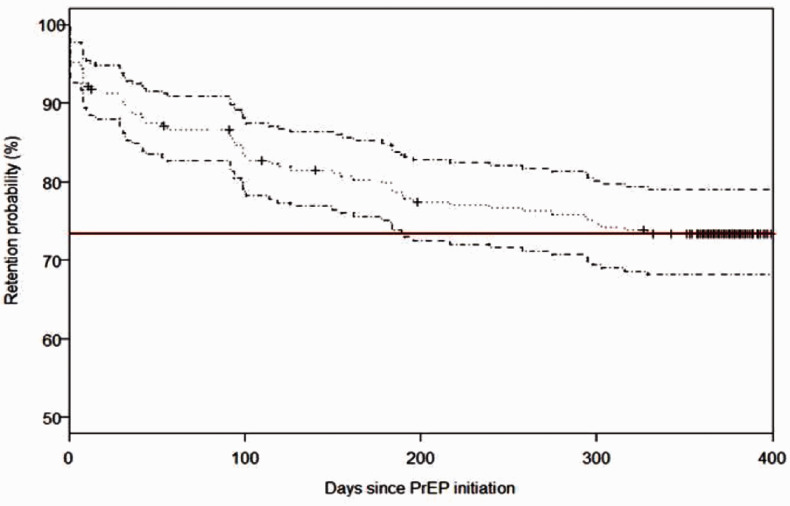
Kaplan–Meier curve for time to PrEP discontinuation. PrEP: pre-exposure prophylaxis. Note: Red line represents the retention probability at 365 days.

Among the 267 participants who were prescribed PrEP, 90.1% were retained in PrEP care at one month (30 days), 79.9% at six months (180 days), and over 2/3 (73.4%) at 12 months (365 days) of follow-up (Figure 2). Reasons for discontinuation that are beyond participants and program control such as death, pregnancy, serious injury, or moving out of the area were right-censored. This information was collected by nurses at the clinic or peer-educators in the field.

The Cox proportional hazard analysis has shown that older age among FSWs was the only significant predictor of lower discontinuation (Table 2). Compared to the 18–24-year age group, the 25–34 (HR= 0.5, 95% CI: 0.2-0.9, 35–44 (HR= 0.3, 95% CI: 0.2-0.7, and 45+ year age groups (HR= 0.2, 95% CI: 0.1-0.5 were significantly less likely to be associated with discontinuation. We did not find significant differences in discontinuation by site, education, registration as sex worker status, condom use, or HIV risk perception measured at baseline.

**Table 2.** PrEP discontinuation.

	PrEP discontinuation		
Characteristics	HR^a^	(95% CI)	P-value
Site			
Diamniadio	1		
Mbao	1.6	(0.8–3.0)	0.160
Pikine	0.8	(0.4–1.7)	0.593
Rufisque	1.3	(0.7–2.5)	0.381
Age (years)			
18–25	1		
26–35	0.5	(0.2–0.9)	0.045
36–45	0.3	(0.2–0.7)	0.005
>45	0.2	(0.1–0.5)	0.002
Registration status			
Registered	1		
Non-registered	1.2	(0.7–1.9)	0.217
Education			
Never been to school	1		
Has been to school	0.7	(0.4–1.1)	0.153
Marital status			
Single	1		
All other (Married/ Separated/ Divorced/Widow)	0.7	(0.4–1.3)	0.261
Risk perception			
Above median	1		
Lower than median	0.8	(0.5–1.3)	0.287
Condom use (during last sex with a client)			
Yes	1		
No	1.2	(0.3–4.9)	0.798

HR: hazard ratio; 95% CI: 95% confidence interval; PrEP: pre-exposure prophylaxis.

^a^Only unadjusted HRs were provided; multivariate models for adjusted HR were not conducted because only age came out significantly associated with discontinuation in the univariate models.

**Figure 3.**
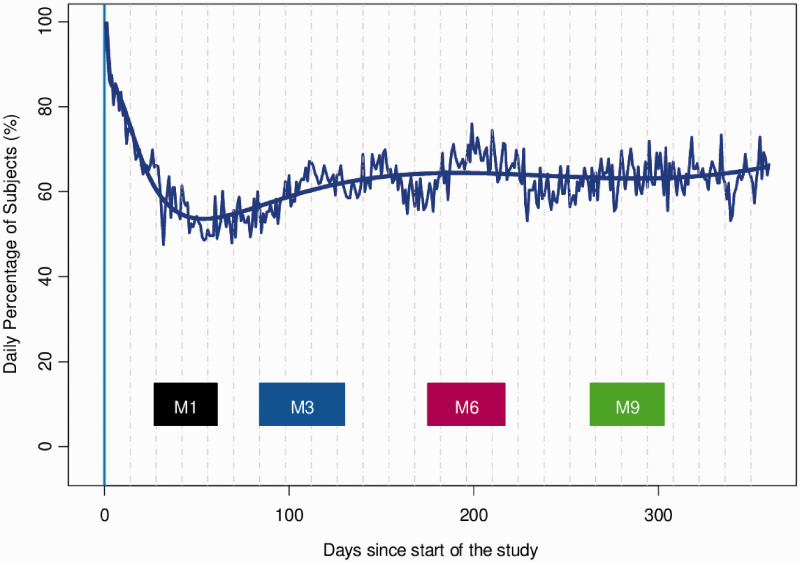
Daily proportion of subjects taking PrEP as prescribed and as measured by MEMS®. (Broken curve = observed proportion, smooth curve = model predictions, rectangle = intervention visits).

Finally, Figure 3 depicts the evolution of the percentage of subjects taking the PrEP medication as prescribed over time. The rectangles in the bottom of the plot correspond to the periods where the intervention visits typically took place. The daily percentage of participants adherent to the prophylactic drug was initially 80% but rapidly dropped to 50% in the first two months. After the first intervention visit, this percentage increased to 65% and remained stable afterwards.

FSWs reported ‘simply forgot’ (20%), ‘too busy with other things’ (18%), and ‘ran out of study pills’ (14%) as the top 3 reasons for non-adherence.

### TFV/FTC drug levels

We measured levels of TFV in 168 plasma samples at 3, 6, 9, and 12 months after PrEP initiation. TFV was detected in 51.8% (95% CI: 44.0–59.5) of samples tested. 32.1% (95% CI: 25.2–39.8) of the samples tested had a TFV level of >35.5 ng/ml consistent with daily dosing of Truvada. Similar results were found for FTC detection (data not shown). Between months 3 and 6 there was a significant decrease in the number of samples with TFV detected (66% versus 43.5%) and samples with TFV levels greater than 35.5 ng/ml (42.6% versus 21.7%) (p < 0.05 by Fisher’s exact tests). There was no significant drop in TFV detected or with TFV levels >35.5 ng/ml, between months 3 and 9 or 12 (data not shown).

Among this high-risk population, there were no HIV-1 or HIV-2 seroconversions during the 12-month study period.

Overall, PrEP was well tolerated, with all adverse events possibly related to the study drug being categorized as grade 1 adverse events (non-serious) based on the Division of AIDS/NIH Adverse Event grading table.^19^ There were three adverse events possibly related to the study drug reported, with no grade 1–4 lab abnormalities reported. These included reports of one woman with diarrhea, one with nausea, and one skin rash/itching. All of them were referred to a physician on site for follow-up and treatment as needed. One case of death unrelated to PrEP was also reported (related to participant’s antecedent of substance abuse).

## Discussion

In this open-label demonstration PrEP study, high interest and enrollment (82.4%) and very good retention rates at 12-month follow-up (73.4%) were seen among FSWs in MoH-run clinics in Dakar, Senegal. No incident HIV-1 or HIV-2 infections were found during the 12-month follow-up period in this study. Based on previous HIV incidence estimation of 14.4 cases per 1000 PYO (95% CI: 10.5–17.0) among FSWs at these clinics, we would have expected ∼4 new infections in the absence of PrEP over the period of observation in this study.^10,13,20^

Unregistered sex workers were significantly more likely to enroll than registered sex workers. This is possibly due to the opportunity for new access to the system for those who do not want to register as sex workers but want to have the access and benefits of regular health care.

Younger age was found to be the only significant predictor of lower PrEP retention. This finding is similar to those observed in previous studies conducted among MSM and transgender women in the US.^21,22^ In our context, older sex workers may have had more exposure to prevention messages offered in public health clinics over time or may have fewer customers and more time to come to the scheduled visits. This could be a concern, knowing that several studies have also found that younger women and sex workers could be at higher risk of HIV infection than their older peers.^23,24^

Our results showed high uptake or interest and very good retention rates, supporting a successful implementation of PrEP among FSWs in Senegal. Other recently conducted PrEP demonstration projects in sub-Saharan Africa have had variable success of uptake, but lower retention rates of FSWs. In Benin, West Africa, uptake was also high at 87.1%, but retention at 58.6% at 12 months was lower than in our study.^25^ In South Africa, uptake was very high at 98%, but retention at 12 months was only 22%.^26^ In Swaziland, only 59% of women were retained at one month after PrEP initiation, while very high self-perceived risk of HIV infection, middle age, and having a partner known to be living with HIV were significant predictors of retention at one month.^27^ In Kenya, retention at one, three, and six months was only at 40.3, 26.3, and 14.0% for FSWs.^28^ Outside the continent, high recruitment and retention levels were also seen in India, with uptake at 97.0% and 16-month retention rate at 93.5%.^29^ However, the India study was conducted within a context of a community-based organization project and not in a government-run public health clinic.^30^

 As seen in similar other PrEP studies among FSWs in Africa, reasons for being lost to follow-up were mostly related to high mobility.^23,24,31^ Some of the most common reasons for being lost to follow-up listed were ‘moved out of the area,’ ‘missed more than 2 visits’ mostly while traveling, and ‘pregnancy.’ The first two reasons cited could have been resolved if PrEP was available in other regions of the country. For the third reason listed, this was due to safety concerns at the time the study was initiated, and pregnant FSWs were given a second consent form with the possibility to make informed decision to stay in the study or not. However, since then research has shown that there are no major safety concerns for prescribing PrEP to pregnant or lactating women.^32,33^ Given the increased risk of HIV infection in FSWs who become pregnant, FSWs on PrEP who became pregnant could have benefited from staying in the study.

In our study, the daily overall percentage of participants adherent to PrEP as measured by MEMS® caps was initially 80% but dropped rapidly to 50% in the first two months; it then rebounded slightly to stay around 65% for the rest of the study. Studies comparing MEMS® data with drug concentrations show that there is 97% accuracy between opening the pharmaceutical package and time of ingestion of the prescribed dose.^34^

The findings on adherence was comparable to what was seen in Benin^25^ and in randomized clinical trials involving high-risk women,^7–9^ but slightly lower than the adherence rates between 70 and 85% reported in South Africa among FSWs.^26^ However, the adherence rates reported in South Africa were based on patient self-reported measures, while this Senegal study used MEMS caps and drug blood level measurements.

Additional details on individual adherence as measured by MEMS® caps and drug blood level and the effect of the intervention will be presented subsequently.

The adherence rates seen in this study may be influenced by the fact that despite being advised to take PrEP daily, participants tended only to take it during times they felt at risk, with ‘PrEP breaks’ during long holiday periods or when visiting parents or relatives in other regions or when not practicing sex work for any other reason.^35,36^ Fourteen percent of participants ran out of pills at some point during the study, mostly related to mobility issues. With no seroconversions among the FSW PrEP users in Senegal, a ‘prevention-effective adherence’ method should be seen as a possible effective way of using PrEP in this population. Although continuous daily PrEP is an effective preventive method, issues such as use during periods of low risk for HIV exposure or the potential combination of other prevention methods such as condoms by FSWs need to be taken into account.^35,36^ Encouragingly, we found no evidence of risk compensation among FSWs on PrEP as measured by self-reported behavior or through Yc-DNA detection.^37^

## Limitations and strengths

One limitation of the study is that no comparison group was included versus the PrEP intervention, limiting the ability to assess the effectiveness of PrEP in addition to existing integrated services. This is because considering the current knowledge on the effectiveness of PrEP, it would not have been ethical to have a comparison group with no access to the medication.

Additionally, the data reported are based on populations of FSWs in urban settings, possibly limiting generalizability; however, we have no indication of having differences between sex workers in urban versus rural settings.

The study also included several strengths such as its ‘real-world’ settings using existing MoH infrastructure. The possibility to measure adherence with MEMS® caps and the availability of drug level testing most likely provided a more accurate assessment of actual FTC/TDF use by the study participants.

Overall, we found evidence of successful implementation of PrEP when offered in MoH-run clinics in Senegal, with high interest and very good retention rates at 12-month follow-up among FSWs. The evidence of successful implementation of PrEP in MoH clinics in Senegal will be key to the planned rollout of PrEP by the MoH in the near future in public health care facilities across the country. Interventions addressing age disparity issues in PrEP retention need to be taken into account for a successful implementation nationwide.

## The Senegal PrEP Demonstration team *(alphabetical order)*

*Bill and Melinda Gates Foundation:* Mary Aikenhead, Josie Presley, and Papa Salif Sow

*Institut de Recherche en Santé de Surveillance Epidémiologique et de Formations:* Mame D. Bousso Bao, Saly Amos Diatta, Ousmane Diouf, Daouda Gueye, Coumba Touré Kane, Moustapha Mané, Aminata Mboup, Souleymane Mboup, Anna Julienne Ndiaye, Birahim Pierre Ndiaye, and Ibrahima Traoré.

*Senegal’s Ministry of Health and government:* Aichatou Barry, Diambogne Ndour, Cheikh Tidiane Ndour, Bouna Sall, Cheikh Saadibou Senghor; Mbaye Thiam, and Safiatou Thiam

*University of Washington, Seattle:* Geoffrey S. Gottlieb, Stephen E. Hawes

*Westat:* Victoria Kioko, Fatima D. Jones, Moussa Sarr, and Carlos Suarez.
